# Antifungal Activity of *Lactobacillus pentosus* ŁOCK 0979 in the Presence of Polyols and Galactosyl-Polyols

**DOI:** 10.1007/s12602-017-9344-0

**Published:** 2017-11-06

**Authors:** Lidia Lipińska, Robert Klewicki, Michał Sójka, Radosław Bonikowski, Dorota Żyżelewicz, Krzysztof Kołodziejczyk, Elżbieta Klewicka

**Affiliations:** 1 Lodz University of Technology, Institute of Fermentation Technology and Microbiology, Wolczanska 171/173, 90-024 Lodz, Poland; 2 Lodz University of Technology, Institute of Food Technology and Analysis, Stefanowskiego 4/10, 90-024 Lodz, Poland; 3 Lodz University of Technology, Institute of General Food Chemistry, Stefanowskiego 4/10, 90-024 Lodz, Poland

**Keywords:** Antifungal activity, Galactosyl-polyols, *Lactobacillus*, Metabolites, Polyols, SEM

## Abstract

The antifungal activity of *Lactobacillus pentosus* ŁOCK 0979 depends both on the culture medium and on the fungal species. In the control medium, the strain exhibited limited antagonistic activity against indicator food-borne molds and yeasts*.* However, the supplementation of the bacterial culture medium with polyols (erythritol, lactitol, maltitol, mannitol, sorbitol, xylitol) or their galactosyl derivatives (gal-erythritol, gal-sorbitol, gal-xylitol) enhanced the antifungal properties of *Lactobacillus pentosus* ŁOCK 0979. Its metabolites were identified and quantified by enzymatic methods, HPLC, UHPLC-MS coupled with QuEChERS, and GC-MS. The presence of polyols and gal-polyols significantly affected the acid metabolite profile of the bacterial culture supernatant. In addition, lactitol and mannitol were used by bacteria as alternative carbon sources. A number of compounds with potential antifungal properties were identified, such as phenyllactic acid, hydroxyphenyllactic acid, and benzoic acid. *Lactobacillus* bacteria cultivated with mannitol synthesized hydroxy-fatty acids, including 2-hydroxy-4-methylpentanoic acid, a well-described antifungal agent. Scanning electron microscopy (SEM) and light microscopy confirmed a strong antifungal effect of *L. pentosus* ŁOCK 0979.

## Introduction

Filamentous fungi and yeasts are present in almost all types of ecosystems due to their high adaptation ability and low nutritional requirements. Filamentous fungi are widespread food spoilage microorganisms responsible for significant economic losses in the agri-food industry [6]; they are also a major health concern due to mycotoxin production. The most common genera of spoilage fungi include *Penicillium*, *Fusarium*, *Aspergillus*, *Cladosporium*, and *Rhizopus* [21]. Commercial foodstuffs are usually protected from such microorganisms by physical and chemical techniques. However, as chemical preservatives have become less socially acceptable, natural preservation methods are being sought. Lactic acid fermentation has been known and used these purposes since antiquity. In recent years, lactic acid bacteria (LAB) have been extensively investigated for their antifungal properties and bioprotective cultures have been proposed as a promising biotechnological approach [22, 24, 25]. Of particular application interest are lactobacilli, which convert carbohydrates into lactic and acetic acids (primary metabolites), as well as a range of secondary metabolites, such as carbon dioxide, ethanol, hydrogen peroxide, fatty acids, acetoin, diacetyl, cyclic dipeptides, bacteriocins, and bacteriocin-like inhibitory substances [3]. Since these metabolites exhibit only weak antifungal properties, many research teams are seeking *Lactobacillus* strains with a higher natural ability to inhibit fungal and yeast growth [4, 9, 14, 15, 26]. Ryu et al. [26] reported that *Lactobacillus plantarum* HD1 synthesizes 5-oxododecanoic acid (MW 214), 3-hydroxydecanoic acid (MW 188), and 3-hydroxy-5-dodecenoic acid (MW 214), which are considered antifungal. In turn, Magnusson [14, 16] showed that some LAB can convert glycerol to 1,3-propanediol, which inhibits fungal growth. While the qualitative and quantitative composition of antifungal compounds generated by LAB is species- or even strain-specific, it can be modulated by culture medium modification. For instance, Lipińska et al. [13] adjusted the antifungal spectrum of lactobacilli by adding polyols and their galactosyl derivatives, proving that the antagonistic activity of LAB depends on culture medium composition, the LAB species, and the sensitivity of the fungal species. It was found that in the presence of xylitol and gal-xylitol in the bacterial culture medium *Lactobacillus pentosus* ŁOCK 0979 effectively inhibited the growth of *A. niger*, *A. alternata*, *A. brassicicola*, *F. lateritium*, and *M. hiemalis* [13]. The modulation of LAB metabolism by supplementing the culture medium with various, often atypical, compounds may give rise to new systems inhibiting the growth of spoilage microorganisms.

The objective of the study was to determine the antifungal properties, metabolite profile, and enzymatic activity of the strain *L. pentosus* ŁOCK 0979 cultured in the presence of polyols, namely, erythritol, xylitol, maltitol, mannitol, sorbitol, and lactitol, and their transglycosylation derivatives (gal-erythritol, gal-xylitol, and gal-sorbitol).

## Materials and Methods

### Microbiological Strains and Polyols

The study material consisted of the bacterial strain *L. pentosus* ŁOCK 0979 and 10 fungal strains deposited with the Pure Cultures Collection of Industrial Microorganisms of the Institute of Fermentation Technology and Microbiology, Lodz University of Technology (ŁOCK 105). The indicator fungi included the yeasts *Candida vini* 0008 and 0009 and the molds *Mucor hiemalis* 0519, *Geotrichum candidum* 0511, *Alternaria alternata* 0409, *Alternaria brassicicola* 0412, *Aspergillus niger* 0433, *Fusarium lateritium* 0508, *Aspergillus ochraceus*, and *Penicillium* sp. Two of the tested fungi, *A. ochraceus* and *Penicillium* sp., were newly isolated from spoiled food.

Fungi were grown in Sabouraud 4% dextrose agar (Merck) and bacteria in MRS medium (Merck) supplemented with 1% (m/v) polyols (erythritol, lactitol, maltitol, mannitol, sorbitol, xylitol) or galactosyl polyols (gal-erythritol, gal-sorbitol, gal-xylitol). The microorganisms were cultured at 30 °C under aerobic conditions. Fungi were stored at 4 °C on Sabouraud dextrose agar slants (Merck), and bacteria were kept at − 20 °C in 20% (*v*/*v*) glycerol.

The polyols used in the study (erythritol, lactitol, maltitol, mannitol, sorbitol, xylitol) occur naturally in some foodstuffs and may be added to others, e.g., as sweeteners. In turn, gal-erythritol, gal-xylitol, and gal-sorbitol are modern prebiotics which confer beneficial effects [8], as related in the blood and digesta of laboratory rats (Klewicki 2007).

### Synthesis of Galactosyl Derivatives of Erythritol, Sorbitol, and Xylitol

Galactosyl derivatives of erythritol, sorbitol, and xylitol were obtained by enzymatic transglycosylation using β-galactosidase EC 3.2.1.23 from *Kluyveromyces lactis* (Novozymes A/S, Bagsvaerd, Denmark). The procedure for galactosyl-xylitol synthesis was described by Klewicki [11].

### Determination of Antifungal Activity of *Lactobacillus pentosus* ŁOCK 0979 in the Presence of Polyols and Galactosyl-Polyols

The antagonistic activity of *L. pentosus* ŁOCK 0979 against the indicator fungi was tested using the double-layer method described by Lipińska et al. [13]. First, 10 μL of overnight bacterial culture was dropped on MRS agar plates (Merck or BTL) supplemented with 1% (*m*/*v*) polyols, galactosyl-polyols, or galactose, separately. The control group consisted of MRS agar plates (Merck) with lactobacilli colonies cultured with neither polyols nor gal-polyols. After 18–24 h, the plates were overlaid with Sabouraud 4% dextrose agar (Merck) inoculated with an indicator fungal strain (10^5^–10^6^ spores × mL^−1^). Indicator strain inhibition zones around *Lactobacillus* sp. colonies were measured after 24–72 h of cultivation at 30 °C. The results were given as fungal inhibition diameters minus the diameter of *Lactobacillus* sp. colonies.

### Preparation of Cell-Free Supernatant After Lactic Acid Fermentation

Following lactic acid fermentation by *L. pentosus* ŁOCK 0979 in media with one of polyols or galactosyl-polyols, samples of cell-free supernatant (CFS) were prepared in order to identify and quantify the antifungal agents produced by the bacteria in the modified MRS media.

The media consisted of MRS broth (Merck) containing 1% (*m*/*v*) glucose supplemented with 1% (*m*/*v*) of one of the polyols (erythritol, lactitol, maltitol, mannitol, sorbitol, or xylitol) or one of the gal-polyols (gal-erythritol, gal-sorbitol, or gal-xylitol). The final pH was 5.7 ± 0.2. In the first step of the experiment, 200 mL of a medium was inoculated with 3% (*v*/*v*) of overnight *L. pentosus* ŁOCK 0979 culture (10^5^–10^6^ cfu × mL^−1^), and incubated for 48 h at 30 °C. Subsequently, the samples were centrifuged (10 min, 12,000×*g*, 20 °C), and the supernatants were filtered using 0.22-μm syringe filters. CFS were stored at − 20 °C for further study.

### Determination of the Content of Polyols and Saccharides Using HPLC

Each CFS sample was diluted 10-fold. The obtained solution was passed through a 5-mL column BAKERBOND® spe Octadecyl (18) (J.T. Baker, USA) with cation and anion exchange resins (1:2 *v*/*v*). The first fraction (3 mL) was discarded, and the second one (3 mL) was collected for HPLC analysis. The content of saccharides and polyols was determined using an Aminex HPX-87C column from Bio-Rad (0.78 × 30 cm, mobile phase: water, flow rate: 0.5 mL × min^−1^, 85 °C). An RI detector and an integrating system from Knauer were used. The tests were done in triplicate and prepared in three parallel columns. Statistical analysis consisted of the Duncan test (*p* ≤ 0.05).

### Spectrophotometric Determination of Glucose

Glucose concentration in the pure culture medium and in post-fermentation CFS was determined spectrophotometrically according to the instructions supplied with the enzymatic kit (BioMaxima) and a calibration curve. CFS and pure culture medium samples were diluted 50- and 100-fold, respectively, relative to the initial glucose content of the medium (10 g × L^−1^). Subsequently, 1 mL of the reagent (glucose oxidase and glucose peroxidase) and 0.01 mL of a tested sample (diluted culture or pure medium) were placed in a cuvette and mixed. After 5 min (37 °C), the absorbance of the tested sample was measured relative to the reagent blank (*λ* = 540 nm), with the results being proportionate to glucose content in the sample. Based on the prepared calibration curve (in the range of 0.01–4 g glucose × L^−1^) glucose concentration was determined both in medium and CFS samples, accounting for their dilution. The tests for each sample were done in triplicate, and statistical analysis involved one-way ANOVA (*p* ≤ 0.05).

### Concentration of d-Lactic Acid, l-Lactic Acid, and Acetic Acid

The quantification of d-lactic, l-lactic, and acetic acid requires enzymatic reactions described in the assay procedures: K-DLLATE 07/14 and K-ACET 11/05 (Megazyme International Ireland). In the case of d- and l-lactic acids, the manufacturer’s procedure for the sequential assay of both optical isomers was applied. The concentration of all tested acids was estimated using colorimetric tests with the absorbance measured (*λ* = 340 nm) in a control sample (non-inoculated medium) and diluted CFS. The calculations were made according to the manufacturer’s recommendations, taking into consideration the dilution factor (F = 50). The tests were done in triplicate, and statistical analysis involved one-way ANOVA (*p* ≤ 0.05).

### Quantification of Antifungal Acids Using UHPLC-MS in Conjunction with QuEChERS

Antifungal metabolites produced by *L. pentosus* ŁOCK 0979 in the presence of polyols and their galactosyl derivatives were quantified using the QuEChERS method and an ultra-high-performance liquid chromatography-mass spectrometry (UHPLC-MS) system according to a protocol modified from Oliveira et al. [20]. In the sample preparation step, 1 mL of formic acid, 10 mL of ethyl acetate, and 10 mL of a CFS sample were added to a Falcon test tube containing 4 mg of magnesium sulfate and 1 g of sodium chloride. The mixture was shaken for 1 min and centrifuged (10 min, 1077×*g*). Then 5 mL of the organic solvent was removed and added to an Agilent dSPE kit (150 mg of C18, 900 mg of magnesium sulfate). The mixture was shaken for 1 min, centrifuged (10 min, 1077×g), and decanted into a test tube with 100 mL of dimethyl sulfoxide (DMSO). The solutions were concentrated for 3.5 h in a ScanVac ScanSpeed 40 centrifuge evaporator (2000 rpm, 45 °C) equipped with a CoolSafe 110-4 Pro cold trap (Labogene, Lynge, Denmark) until only 100 μL DMSO remained. The concentrated solution was mixed with 400 μL of 10% (*v*/*v*) acetonitrile, centrifuged (10 min, 10,000×*g*), and transferred into a 1.5-mL amber vial.

The following 13 antifungal compounds were quantified: dl-3-phenyllactic acid, dl-p-hydroxyphenyllactic acid, benzoic acid, hydrocaffeic acid, hydrocinnamic acid, vanillic acid, 4-hydroxybenzoic acid, catechol, caffeic acid, ferulic acid, 3-hydroxybenzoic acid, 2,4-dihydroxybenzoic acid, and p-coumaric acid.

A Dionex UltiMate 3000 ultra-high-performance liquid chromatograph from Thermo Fisher Scientific (Germering, Germany) coupled with a diode array detector (DAD) and a Q Exactive Orbitrap mass spectrometer (MS, Thermo Fisher Scientific, Bremen, Germany) was used for LC-MS analysis. Chromatographic separation was performed using a 150-mm C18 column with a 2.1-mm internal diameter and 2.6-μm particle size (Kinetex 2.6u, Torrance, CA, USA). The column temperature was maintained at 30 °C, and the injection volume was 2.5 μL. The mobile phase consisted of the following: A was water containing 0.1% formic acid and B was a mixture of acetonitrile and water (90:10, *v*/*v*) containing 0.1% formic acid. The flow rate was 0.5 mL/min. The following gradient was used: 0–16.5 min, 5–40% B; 16.5–17.5 min, 40–95% B; 17.5–20 min, 95% B; 20–22 min, 95–5% B; 22–27 min, 5% B. After DAD detection, the separated compounds entered into the MS system via a heated electrospray ionization (H-ESI) source with a flow rate of 0.5 mL/min. Analyses were carried out in the negative ion mode. Chromatographic data were collected using Xcalibur software (Thermo). The source parameters were as follows: a vaporizer temperature of 400 °C, an ion spray voltage of 4 kV, a capillary temperature of 380 °C, and sheath and auxiliary gas flow rates of 60 and 15 units, respectively. The detector was operated in either full MS or full MS/dd-MS2 scan modes. In the full MS mode, the scan rage of *m/z* 50–400 was used. The full MS/dd-MS^2^ scan mode was used to generate MS2 data. In this mode, the selected precursor ions entered into a high-energy collision-induced dissociation (CID) cell, where they were fragmented with normalized collision energy (NCE) to obtain product ion spectra (MS^2^). In our experiments, the NCE used to generate MS^2^ spectra was set to 30. Tuning and optimization were performed using direct injection of the standard solution diluted in an 80:20 (*v*/*v*) mixture of mobile phases A and B at a flow rate of 0.25 mL/min. Acids were quantified using the selected ion monitoring (SIM) mode. The standard curves of these compounds were used for quantification. Table 1 gives acquisition parameters for 13 acids in the tested solution.

**Table 1:** LC-MS acquisition parameters for acids detected in post-fermentation cell-free supernatant

Compound	Retention time (min)	Confirmation ion (*m/z*)	Quantitation ion (*m/z*)
Catechol	3.27	–	109
dl-*p*-Hydroxyphenyllactic acid	3.44	163, 135, 119	181
3-Hydroxybenzoic acid	4.38	93	137
Hydrocaffeic acid	4.88	137, 109	181
2,4-Dihydroxybenzoic acid	5.47	109	153
4-Hydroxybenzoic acid	5.47	93	137
Vanillic acid	5.52	152, 123, 108	167
Caffeic acid	5.88	135	179
dl-3-Phenyllactic acid	6.80	147, 119	165
*p*-Coumaric acid	7.88	119	163
Ferulic acid	8.74	178, 134	193
Hydrocinnamic acid	12.17	121	149

Acid quantification was performed in triplicate, and statistical analysis was conducted using the Duncan test (*p* ≤ 0.05).

### Identification of Fatty Acids and Hydroxylated Fatty Acids by Gas Chromatography Coupled with Mass Spectrometry

*Lactobacillus pentosus* ŁOCK 0979 was grown at 30 °C in 150 mL of MRS broth (Merck) with 1% (*m*/*v*) mannitol. The 48-h culture was centrifuged (10 min, 12,000×*g*, 20 °C), and supernatant pH was adjusted to 4.0 with hydrochloric acid. Then, 100 mL of the sample was extracted with 30 mL of dichloromethane, mixed for 3 min, and settled for 10 min. Extraction of the aqueous phase was repeated twice using further portions of dichloromethane. The organic phases were combined, dried over anhydrous sodium sulfate, and, following filtration, concentrated to approx. 0.5 mL in a rotatory evaporator. The residue was derivatized with 200 μL of 0.25 M trimethylsulfonium hydroxide solution (TMSH, Sigma Aldrich) in methanol.

The samples were analyzed by gas chromatography coupled with mass spectrometry (TRACE GC Ultra—ISQ) using a Stabilwax-DA capillary column (30 m × 0.25 mm i.d., film thickness 0.25 μm). The operating conditions were as follows: temperature program—50 °C (3 min)–240 °C (30 min) at 4 °C/min, injection temperature—240 °C, carrier gas—helium (constant flow 1 mL/min). Mass spectrometer parameters were as follows: 33–550 amu, ionization energy 70 eV, ion source temperature 200 °C. Identification of compounds was based on a comparison of their mass spectra with computerized libraries (Wiley Registry 10th Edition/NIST Mass Spectral Library 2012).

Mannitol was chosen as one of the best agents enhancing the antifungal effect of *L. pentosus* ŁOCK 0979. Moreover, in the presence of mannitol, many signals from acidic compounds were obtained using UHPLC-MS analysis, but they could not be identified by the UHPLC-MS method due to their molecular structure (data not presented).

### API®ZYM 25200 Test of Bacterial Enzymatic Activity

Bacteria were grown for 24 h in 9 mL of MRS broth (Merck) with 1% (*m*/*v*) polyols or galactosyl-polyols, added one by one. The cultures were centrifuged (10 min, 12,000×*g*, 20 °C), and the biomass was suspended in saline to obtain a cell concentration corresponding to 5–6 on the McFarland scale (approx. 1.5 × 10^9^ cells × mL^−1^). API ZYM strips were placed in API ZYM boxes humidified by distilled water. The strips were inoculated with 65 μL of the sample and incubated for 4 h at 37 °C. Then, the reagents ZYM A and ZYM B (bioMerieux) were added dropwise. The strips were placed under a powerful light source for 10 s and then exposed to daylight for 5–10 min. Results were read according to the manufacturer’s recommendations.

### The Effects of Cell-Free Supernatants on Fungal Growth and Morphology Evaluated with Scanning Electron Microscopy and Light Microscopy

Fungal strains sensitive to the metabolites of polyols or gal-polyols were selected based on the antagonistic activity of *L. pentosus* ŁOCK 0979 cultured in different culture media. CFS samples were added to Sabouraud 4% dextrose agar (Merck) in the amount of 10% (*v*/*v*). The medium was subsequently placed in 6-well plates and inoculated with selected fungal strains using an inoculation loop. Microscopic examination was carried out after 2 days (yeasts) and 7 days (molds) using a light microscope. Additionally, yeast morphology was examined using a scanning electron microscope (JEOL JCM-6000, Tokyo, Japan) after coating with gold particles for 45 s (JEOL JFC-1200 Fine Coater, Tokyo, Japan). The experiments were conducted in duplicate. The control samples consisted of fungi cultured on Sabouraud agar without bacterial CFS.

## Results

### Antifungal Activity of *Lactobacillus pentosus* ŁOCK 0979 in the Presence of Polyols and Galactosyl-Polyols

The antagonistic activity of *L. pentosus* ŁOCK 0979 against the tested yeasts was weak, but its anticandidal properties were enhanced in the presence of galactosyl-polyols, and especially gal-erythritol. The addition of gal-sorbitol and gal-xylitol to the bacterial culture medium led to inhibition of only one of the two strains of yeast, that is, *C. vini* ŁOCK 0009 (Table 2).

**Table 2:** Antifungal activity of *Lactobacillus pentosus* ŁOCK 0979

Growth media	Yeasts		Molds							
	1	2	3	4	5	6	7	8	9	10
MRS (control)	**–**	**–**	**+**	**+**	**+++**	**–**	**+**	**–**	**+++**	**+++**
MRS + erythritol	–	–	+	+	++	+	++	+	++	+++
MRS + lactitol	–	–	–	–	+++	++	+	++	+++	+++
MRS + xylitol	–	–	+	–	+	++	++	+	+++	+++
MRS + maltitol	–	–	+	–	+++		+++	++	++	+++
MRS + mannitol	–	–	+	–	+++	+++	++	+	+++	+++
MRS + sorbitol	–	–	+	–	+++	–	+++	–	+++	++
MRS + gal-erythritol	++	+	+/−	–	++	+++	++	–	+++	+++
MRS + gal-xylitol	–	+/−	+	+/−	+++	+++	+++	+/−	+++	+++
MRS + gal-sorbitol	–	+	+	–	+++	+++	+++	+/−	X	+++
MRS + galactose	–	–	+	–	+++	+++	+++	–	+	+
MRS + galactose (glucose-free)	–	–	+	–	++	++	++	–	+	+

1. *C. vini* 0008, 2. *C. vini* 0009, 3. *M. hiemalis*, 4. *G. candidum*, 5. *A. alternata*, 6. *A. brassicicola*, 7. *F. lateritium*, 8. *A. niger*, 9. *A. ochraceus*, 10. *Penicillium* sp.

*X* no tested, *−* no inhibition zone, *+/−* inhibition zone between 0.5 and 2 mm, *+* inhibition zone between 2.1 and 10 mm, *++* inhibition zone between 10.1 and 20 mm, *+++* inhibition zone above 20 mm, *nt* not tested

Mold inhibition by *L. pentosus* ŁOCK 0979 depended both on the culture medium composition and on mold species. The *A. alternata* test strain and the *A. ochraceus* and *Penicillium* sp. strains isolated from the environment exhibited the greatest sensitivity to lactic acid fermentation products both in the controls and in samples with polyols and gal-polyols (Table 2). In contrast, the growth of *A. brassicicola* and *A. niger* was inhibited by *L. pentosus* ŁOCK 0979 only if the bacterial culture medium was supplemented with polyols (both mold strains) or gal-polyols (only *A. brassicicola*). The antifungal activity of *L. pentosus* ŁOCK 0979 was also improved by polyols and their galactosyl derivatives with respect to *F. lateritium.* Its growth was inhibited by the bacteria cultured in the presence of maltitol and sorbitol, as well as all tested galactosyl-polyols. Antagonistic activity against *G. candidum* and *M. hiemalis* was weak (Table 2).

In a similar way, additional control trials were conducted using MRS medium with glucose (Merck) and 1% (*m*/*v*) galactose, as well as a glucose-free MRS medium (BTL) with 1% (*m*/*v*) galactose. These media enhanced the antagonistic activity of *L. pentosus* ŁOCK 0979 only against one indicator fungal strain (*F. lateritium*) as compared to bacteria cultivated on MRS agar (Merck)*.* Therefore, it can be assumed that the small amounts of galactose released as a result of galactosyl-polyol hydrolysis (Table 3) are not a critical determinant of antifungal properties.

**Table 3:** Concentration of polyols and saccharides before and after lactic acid fermentation by *Lactobacillus pentosus* ŁOCK 0979 in media containing polyols and their galactosyl derivatives

Media	Compounds	Initial content (g × L^−1^)	Residual content (g × L^−1^)
MRS (control)	Glucose	20.9 ± 3.61	–
MRS + erythritol	Glucose	25.4 ± 0.01	–
	Erythritol	10.6 ± 0.27a	10.1 ± 0.10a
MRS + lactitol	Glucose	20.2 ± 0	–
	Lactitol	10.0 ± 0a	9.03 ± 0.11b
MRS + xylitol	Glucose	18.7 ± 0.92	–
	Xylitol	10.3 ± 0.10a	10.0 ± 0a
MRS + maltitol	Glucose	20.2 ± 0.77	–
	Maltitol	10.4 ± 0.54a	10.0 ± 0a
MRS + mannitol	Glucose	13.14 ± 0.11	–
	Mannitol	10.0 ± 0.04a	7.4 ± 0.70b
MRS + sorbitol	Glucose	19.0 ± 0.34	–
	Sorbitol	10.0 ± 0a	9.7 ± 0.22a
MRS + gal-erythritol	Glucose	18.9 ± 0.01	–
	Gal-erythritol	10.03 ± 0a	5.8 ± 0.73b
	Erythritol	–	1.7 ± 0.09
	Galactose	–	0.6 ± 0.09
MRS + gal-xylitol	Glucose	20.6 ± 0	–
	Gal-xylitol	9.9 ± 0.68a	7.7 ± 0.21b
	Xylitol	–	1.0 ± 0.07
MRS + gal-sorbitol	Glucose	18.7 ± 0	–
	Gal-sorbitol	10.5 ± 0.07a	7.8 ± 0.38b
	Sorbitol	–	0.7 ± 0.08
	Galactose	–	0.1 ± 0.02

Means designated with the same lowercase letter are not significantly different (Duncan’s multiple range test)

*–* below the limit of detection

### Content of Polyols and Saccharides in Cell-Free Supernatant

The content of polyols and saccharides before and after lactic acid fermentation by *L. pentosus* ŁOCK 0979 in the presence of polyols and gal-polyols was determined using HPLC, and the concentration of glucose was evaluated spectrophotometrically (Table 3). The results show that in lactic acid fermentation the bacteria used glucose as a primary carbon source, while the galactosyl-polyols and polyols (lactitol, mannitol) were used as additional carbon sources to varying degrees (Table 3).

The mannitol and lactitol present in the culture media were partially used by *L. pentosus* ŁOCK 0979 (as reflected in 10 and 26% decline in concentration after lactic acid fermentation, respectively). Galactosyl-polyols (gal-erythritol, gal-xylitol, and gal-sorbitol) were hydrolyzed to galactose and the respective polyols. Residual galactose was found in post-fermentation CFS from samples supplemented with gal-erythritol and gal-sorbitol (Table 3). The content of erythritol, xylitol, maltitol, and sorbitol in the medium did not change significantly following lactic acid fermentation (Table 3).

### Acidity and Production of Acetic and Lactic Acids

The concentration of acetic acid and lactic acid (l- and d-enantiomers separately) was evaluated enzymatically using Megazyme kits. The total content of acetic acid was from 4.09 ± 0.178 to 7.62 ± 0.010 g × L^−1^, while that of lactic acid was from 4.58 ± 0.390 to 20.26 ± 1.489 g × L^−1^; in all samples, the dominant stereoisomer was l-lactic acid accounting for 61–100% of the total (Table 4). The mean pH of the post-fermentation supernatant was higher in the case of gal-polyols (pH 4.13 ± 0.12) than polyols (pH 3.88 ± 0.290), except for sorbitol (pH 4.4). Samples with higher pH (gal-polyols, sorbitol) exhibited a lower concentration of lactic acid (4.58–9.49 g × L^−1^) and a higher concentration of acetic acid (5.76–7.62 g × L^−1^). *L. pentosus* ŁOCK 0979 generated the highest amounts of lactic and acetic acids in the presence of xylitol (20.26 ± 1.489 g × L^−1^) and gal-xylitol (7.62 ± 0.010 g × L^−1^), respectively (Table 4).

**Table 4:** Production of acetic, d-lactic, and l-lactic acids by *Lactobacillus pentosus* ŁOCK 0979 in the presence of polyols and gal-polyols

Medium	pH	Production of lactic acid			Production of acetic acid (g × L^−1^)
		dl-Lactic acid (g × L^−1^)	% d-lactic acid [%]	% l-lactic acid [%]	
Non-inoculated medium MRS	5.7 ± 0.2	–	–	–	0.07 ± 0.000
MRS (control)	3.6	13.89 ± 0.448	7.0	93.0	4.39 ± 0.105
MRS + erythritol	3.7	12.77 ± 0.015	0.0	100.0	4.75 ± 0.178
MRS + lactitol	3.7	15.01 ± 3.458	6.6	93.3	4.09 ± 0.178^a^
MRS + xylitol	4.0	20.26 ± 1.489	14.2	85.8	5.71 ± 0.024^a^
MRS + maltitol	3.9	10.89 ± 0.000	0.0	100.0	4.87 ± 0.003
MRS + mannitol	3.6	15.26 ± 1.619	5.1	94.9	5.76 ± 0.020^a^
MRS + sorbitol	4.4	5.74 ± 1.257	34.8	65,2	6.52 ± 0.010^a^
MRS + gal-erythritol	4.2	9.49 ± 0.234	37.7	62.3	6.61 ± 0.023^a^
MRS + gal-xylitol	4.2	8.81 ± 1.685	38.6	61.4	7.62 ± 0.010^a^
MRS + gal-sorbitol	4.0	4.58 ± 0.390	31.9	68.1	7.17 ± 0.020^a^

^a^Significantly different from the control test

### Effects of Polyols and Gal-Polyols on the Content of Antifungal Acids

The following 13 antifungal acids were quantified: dl-3-phenyllactic acid (PLA), dl-p-hydroxyphenyllactic acid (HPLA), benzoic acid (BA), hydrocaffeic acid (HCaA), hydrocinnamic acid (HCiA), vanillic acid (VA), 4-hydroxybenzoic acid (4-HBA), catechol (Cat), caffeic acid (CaA), ferulic acid (FA), 3-hydroxybenzoic acid (3-HBA), 2,4-dihydroxybenzoic acid (2,4-dHBA), and p-coumaric acid (p-CoumA), using a UHPLC-MS system coupled with QuEChERS. It was found that the minimum inhibitory concentrations of the tested acids are many times higher than their actual concentrations in CFS (Table 5). Statistically significant differences in the content of PLA and HCaA were linked to the composition of the culture medium. PLA content in CFS from the bacterial culture with gal-xylitol differed from that found in cultures with lactitol and xylitol. In the presence of gal-erythritol and gal-xylitol, *L. pentosus* ŁOCK 0979 produced double to triple the amount of vanillic acid and half the amount of Cat as compared to the other CFS. The content of HCaA, HCiA, VA, 4-HBA, Cat, CaA, FA, 3-HBA, 2,4-dHBA, and p-CoumA was low and ranged from approx. 0 (<LOD) to 0.161 mg × L^−1^ (Table 5).

**Table 5:** Content of antifungal acids produced by *Lactobacillus pentosus* ŁOCK 0979 as determined by HPLC coupled with QuEChERS

Polyols in medium	Production of antifungal acids and their referential MIC (mg × L^−1^)												
	PLA	HPLA	BA*	3-HBA*	HCaA	2,4-dHBA	Cat	4-HBA	Vanillic acid	CaA	p-CoumA	FA	HCiA
MRS—non-inoculated	0.107 ± 0.001	0.033 ± 0.003	0.339 ± 0.010	0.062 ± 0.002	0.006 ± 0.001	0.031 ± 0.001	0.015 ± 0.002	< 0.008	0.186 ± 0.006	0.002 ± 0	0.012 ± 0.001	0.009 ± 0.001	< 0.017
MRS (control)	41.513 ± 1.175	4.255 ± 0.069	0.623 ± 0.049	0.072 ± 0.002	0.090 ± 0.001	0.012 ± 0.002	0.045 ± 0.003	< 0.008	0.003 ± 0	0.005 ± 0.001	0.009 ± 0.0	0.008 ± 0.001	< 0.017
Erythritol	40.319 ± 0.865	4.132 ± 0.045	0.614 ± 0.103	0.072.002	0.062 ± 0	0.010 ± 0	0.037 ± 0	< 0.008	0.086 ± 0.007	< 0.002	0.005 ± 0.002	0.008 ± 0.001	< 0.017
Lactitol	51.228 ± 0.482 x	4.872 ± 0.161	0.504 ± 0.022	0.078 ± 0.001	0.088 ± 0.001	0.011 ± 0.001	0.048 ± 0.004	< 0.008	0.070 ± 0.003	< 0.002	0.006 ± 0.001	0.008 ± 0.002	< 0.017
Xylitol	49.988 ± 1.026z	4.869 ± 0.052	0.504 ± 0.017	0.079 ± 0.001	0.083 ± 0.003	0.011 ± 0.001	0.039 ± 0.003	< 0.008	0.075 ± 0.007	< 0.002	0.006 ± 0	< 0.010	< 0.017
Maltitol	24.224 ± 16.272	2.844 ± 1405	0.249 ± 0.094	0.050 ± 0.023	0.060 ± 0.033	0.009 ± 0.002	0.034 ± 0.007	< 0.008	0.041 ± 0.015	0.005 ± 0	0.006 ± 0	0.005 ± 0.003	< 0.017
Mannitol	40.992 ± 0.875	4.128 ± 0.096	0.582 ± 0.007	0.073 ± 0.001	0.085 ± 0.003	0.011 ± 0.001	0.049 ± 0.008	< 0.008	0.086 ± 0.003	< 0.002	0.005 ± 0.002	0.009 ± 0.001	< 0.017
Sorbitol	31.962 ± 14.709	3.93 ± 0.852	0.49 ± 0.282	0.06 ± 0.017	0.06 ± 0.032	0.01 ± 0.002	0.040 ± 0.003	< 0.008	0.084 ± 0.012	0.003 ± 0	0.005 ± 0.001	0.009 ± 0.003	< 0.017
Gal-erythritol	21.91 ± 5.455	6.13 ± 4.311	0.03 ± 0.010	0.06 ± 0.013	0.04 ± 0.010	0.02 ± 0.001	0.018 ± 0.005	< 0.008	0.161 ± 0.057	0.002 ± 0	0.005 ± 0	0.008 ± 0.002	< 0.017
Gal-xylitol	16.65 ± 1.679 X, Z	2.46 ± 0.217	0.34 ± 0.005	0.05 ± 0.005	0.03 ± 0.003 X	0.02 ± 0.001	0.017 ± 0.001	0.012 ± 0.001	0.127 ± 0.001	0.002 ± 0	0.004 ± 0	0.007 ± 0.001	< 0.017
Gal-sorbitol	24.89 ± 8.188	3.14 ± 0.753	0.40 ± 0.106	0.06 ± 0	0.04 ± 0.018	0.01 ± 0.005	0.044 ± 0.001	< 0.008	0.005 ± 0	< 0.002	0.006 ± 0	0.007 ± 0.001	< 0.017
MIC (Oliveira et al. 2015)	7500–10,000	–	20–2000	–	> 10,000	–	> 1000	> 1000	> 100	> 100–2000	> 100	> 100–2000	100–1000

Distributions with both a capital and small letter are significantly different from each other (Tukey’s test *p* ≤ 0.05)

*PLA* dl-3-phenyllactic acid, *HPLA* dl-p-hydroxyphenyllactic acid, *BA* benzoic acid, *3-HBA* 3-hydroxybenzoic acid, *HCaA* hydrocaffeic acid, *2,4-dHBA* 2,4-dihydroxybenzoic acid, *Cat* catechol, *4-HBA* 4-hydroxybenzoic acid, *VA* vanillic acid, *CaA* caffeic acid, *p-CoumA* p-coumaric acid, *FA* ferulic acid, *HCiA* hydrocinnamic acid

*p value estimated in Tukey’s test ≤ 0.05 (significance of differences may not be estimated)

### Production of Hydroxy Fatty Acids in the Presence of Mannitol

Volatile compounds with potential antifungal properties (fatty acids, hydroxy fatty acids) synthesized by *L. pentosus* ŁOCK 0979 cultured in MRS and in the presence of 1% (*m*/*v*) mannitol were identified (Table 6). Mannitol induced antagonistic activity of *L. pentosus* ŁOCK 0979 against some of the test fungi (*A. brassicicola*, *A. niger*, *F. lateritium*), which must therefore be attributable to one or more metabolites synthesized in the presence of this polyol. Moreover, CFS samples revealed 2-hydroxy-4-methylpentanoic acid, a compound described by Ndagano et al. [19] as a strong antifungal agent.

**Table 6:** Volatile compounds produced by *Lactobacillus pentosus* ŁOCK 0979 in MRS broth and in the presence of mannitol

No. Compound		Media		
		MRS—non-inoculated	MRS (control)	MRS + mannitol
1	Butanoic acid	+	+	+
2	2-Methylbutanoic acid	+	+	–
3	Isovaleric acid	+	+	+
4	Caproic acid	+	+	+
5	3-Hydroxypropanoic acid	–	+	+
7	2-Hydroxypropanoic acid	+	+	+
8	2-Hydroxyisocaproic acid	–	+	+
9	Octanoic acid	+	+	+
10	2-Hydroxy-3-methylbutyric acid	–	+	+
11	2-Hydroxybutanoic acid	+	+	–
12	2-Methylthioacetic acid	+	+	–
13	Acetic acid	+	+	–
14	Acetoxyacetic acid	+	–	–
17	2-Hydroxy-3-methylpentanoic acid	–	+	–
18	2-Hydroxy-4-methylpentanoic acid	–	+	+
19	3-(Methylthio)propanoic acid	–	+	–
20	3-Hydroxypropanoic acid	–	+	–
21	Decanoic acid	+	–	–
22	Butanedioic acid	+	–	+
23	2-Oxopentanedioic acid	–	+	–
24	Benzoic acid	+	+	+
25	2-Hydroxypropanoic acid	+	–	+
27	Phenylacetic acid	+	+	+
28	Dodecanoic acid	–	+	+
29	Hydrocinnamic acid	–	+	–
30	Myristic acid	+	+	+
32	Pentadecanoic acid	–	+	–
33	Azelaic acid, dimethyl ester	+	+	–
34	Palmitic acid	+	+	+
35	2-Pyrrolidone-5-carboxylic acid	–	–	+
36	Palmitoleic acid	+	+	+
37	2-Hydroxybenzenepropanoic acid	–	+	+
38	Heptadecanoic acid (C17:0)	+	+	–
39	Citric acid, trimethyl ester	+	–	–
40	Methyl stearate	+	+	+
37	9-Octadecenoic acid	+	+	+
38	11-Octadecenoic acid	–	+	–
39	Linoleic acid	–	+	+
40	9,12-Octadecadienoic acid	+	–	–
37	Linolelaidic acid	–	–	+
38	9,12-Octadecadienoic acid	+	+	+
39	Eicosanoic acid	+	+	–
40	11-Eicosenoic acid	–	–	+
41	3-Octyloxiraneoctanoic acid	+	+	+
42	10-hydroxyoctadecanoic acid	–	+	–

### Enzymatic Activity of *Lactobacillus pentosus* ŁOCK 0979

Examination of the activity of enzymes metabolizing lipids, proteins, and phosphates revealed some minor differences between *L. pentosus* ŁOCK 0979 cultures conducted in media supplemented with various polyols and gal-polyols (Table 7). As compared to the controls, esterase activity was found only in the presence of lactitol and gal-sorbitol, while that of esterase lipase in the presence of gal-erythritol.

**Table 7:** Effects of culture medium on the enzymatic activity of *Lactobacillus pentosus* 0979

Enzymes	Enzymatic activity of *Lactobacillus pentosus* ŁOCK 0979 cultured with polyols and gal-polyols									
	MRS (control)	MRS + erythritol	MRS + lactitol	MRS + xylitol	MRS + maltitol	MRS + mannitol	MRS + sorbitol	MRS + gal-erythritol	MRS + gal-xylitol	MRS + gal-sorbitol
Control	–	–	–	–	–	–	–	–	–	–
Alkaline phosphatase EC	–	–	–	–	–	–	–	–	–	–
Esterase (C4)	–	–	+/−	–	–	–	–	–	–	+/−
Esterase Lipase (C8)	–	–	–	–	–	–	–	+	–	–
Lipase (C14)	–	–	–	–	–	–	–	–	–	–
Leucine arylamidase	+	+	+	+	+	+	+	+	+	+
Valine arylamidase	+	+	+	+	+	+	+	+	+	+
Cystine arylamidase	–	–	–		–	–	–	–	–	–
Trypsin	–	–	–	–	–	–	–	–	–	–
α-Chymotrypsin	–	–	–	–	–	–	–	–	–	–
Acid phosphatase	+/−	+	+	–	+	+	–	+	–	+
Naphthol-AS-BI-phosphohydrolase	–	–	–	–	–	–	–	–	–	–
α-Galactosidase	+	+	+	+	+	+	+/−	+	+	+
β-Galactosidase	+	+	+	+	+	+	+	+	+	+
β-Glucuronidase	+	+	–	+	+	+	+	+	+/−	+
α-Glucosidase	–	+	+/−	+/−	–	–	–	+	–	++
β-Glucosidase	+	+	+	+	+	+	+	+	+	+
N-Acetyl-β-glucosaminidase	–	–	–	–	–	–	–	–	–	–
α-Mannosidase	–	–	–	–	–	–	–	–	–	–
α-Fucosidase	–	–	–	–	–	–	–	–	–	–

### Effects of Polyols and Gal-Polyols on Fungal Growth and Morphology

The yeasts *C. vini* ŁOCK 0008 and ŁOCK 0009 and the mold *A. brassicicola* were examined microscopically following culture in Sabouraud agar with 10% (*v*/*v*) CFS. The results are given in Tables 8 and 10. Scanning electron micrographs of *C. vini* ŁOCK 0009 cultivated in the presence of CFSs are presented in Table 9.

**Table 8.**
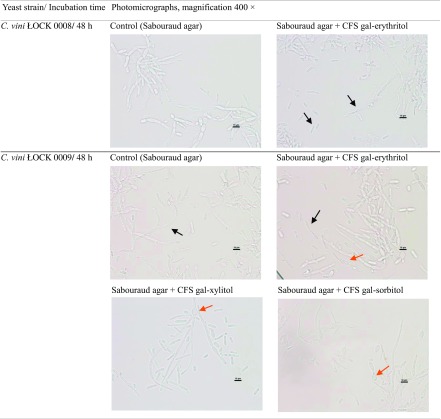
Micrographs of yeasts grown for 2 days in Sabouraud medium with 10% (*v*/*v*) cell-free supernatant of *Lactobacillus pentosus* ŁOCK 0979 cultivated 48 h in the media containing gal-polyols

**Table 9.**
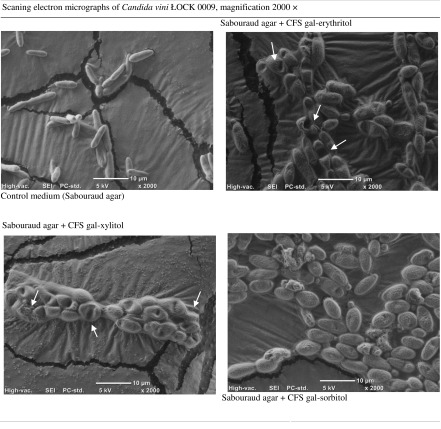
Scanning electron micrographs of yeasts grown for 2 days on Sabouraud agar medium with 10% (*v*/*v*) CFS from 48 h culture of *Lactobacillus pentosus* ŁOCK 0979 in media containing gal-polyols

Light microscopy revealed greater cell differentiation in the yeast *C. vini* ŁOCK 0008 grown in Sabouraud agar with 10% (*v*/*v*) CFS from *L. pentosus* ŁOCK 0979 cultured in the presence of gal-erythritol than the control (Table 8). *Candida vini* ŁOCK 0008 cells were at different developmental stages and included both single cells and some initial degrees of pseudomycelium formation. The same was true of *C. vini* ŁOCK 0009 grown in Sabouraud agar with 10% (*v*/*v*) CFS from *L. pentosus* ŁOCK 0979 cultured in the presence of gal-polyols (gal-erythritol, gal-xylitol, gal-sorbitol). What is more, the addition of gal-polyols to the bacterial medium led to fungal deformation and gave rise to blastoconidia (CFS gal-erythritol, CFS gal-xylitol, CFS gal-sorbitol) (red arrows in the Table 8). The yeast cells were narrower, and some of them became pear-shaped as compared to the controls (Table 8). SEM provided more details about the form and surface of *C. vini* ŁOCK 0009. In the control, yeasts were elliptical and developed pseudohyphae with smooth and flat surfaces (Table 9). The cells of *C. vini* ŁOCK 0009 cultivated with the CFS of *L. pentosus* ŁOCK 0979 in the presence of gal-polyols were strongly deformed. Their shape was warped and the surface rough, and cell damage was visible in the form of concave areas on the surface (Table 9). Additionally, in the presence of CFS gal-xylitol yeasts, cells were coated by extracellular matrix (Table 9).

Morphological changes were also observed in the mycelium of the mold *A. brassicicola* grown in Sabouraud agar with 10% (*v*/*v*) CFS from *L. pentosus* ŁOCK 0979 cultured in bacterial media supplemented with erythritol and xylitol. Furthermore, growth inhibition and mycelium deformation were found for all gal-polyols in the bacterial medium (Table 10). The addition of lactitol and mannitol to the bacterial medium led to complete inhibition of fungal growth (hence no photomicrographs).

**Table 10.**
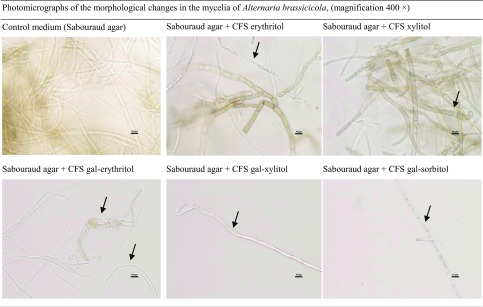
Morphological changes in the mycelia of *Alternaria brassicicola* grown for 7 days in Sabouraud medium with 10% (*v*/*v*) CFS from 48 h cultures of *L. pentosus* ŁOCK 0979 in media containing polyols and gal-polyols

## Discussion

The control of spoilage microorganisms and, by the same token, the extension of the shelf-life of foodstuffs still pose formidable challenges. While the use of *Lactobacillus* sp. as natural bioprotective agents was already reported by Magnusson [14], a definitive explanation of their mechanism of action against undesirable fungi was not provided.

Lactobacilli inhibit the growth of other bacteria (of the same or different species) as well as that of fungi, including pathogenic and toxin-producing molds [1, 7]. While many authors have reported the antifungal properties of LAB [3, 10], their exact underlying mechanisms remain elusive. Nevertheless, it is known that a major role is played by some bacterial metabolites, and especially by organic acids, hydroxy fatty acids, cyclic dipeptides, and low molecular weight proteinaceous compounds [22]. In addition to primary metabolites (lactic and acetic acids), which are produced by all *Lactobacilli.* sp., some LAB species synthesize secondary metabolites, which may selectively affect other microorganisms; these include propionic, hexanoic, salicylic, succinic, formic, 2-pyrrolidone-5-carboxylic, 3-phenyllactic, and 4-hydroxyphenyllactic acids [2, 14, 17]. One of the best described antifungal products of lactic acid fermentation is 3-phenyllactic acid (PLA), synthesized by LAB such as *L. casei*, *L. fermentum*, *L. rhamnosus*, *L. reuteri*, and *L. sakei* [14, 17, 18]. While *L. pentosus* ŁOCK 0979 does produce PLA, its concentration in the CFS is much lower than its minimum inhibitory concentration reported by other authors [20].

The antifungal metabolites of *Lactobacillus* sp. constitute a rich mixture of active compounds whose qualitative and quantitative composition largely depends on the compounds found in the bacterial culture medium. Ndagano et al. [19], who evaluated the effects of different concentrations and proportions of acetic and lactic acids on fungal viability, observed significant synergies: the mixture was more potent than the sum of its individual components taken together. Synergies may also be found for some parameters of the culture medium, such as pH.

The study presented herein was preceded by evaluation of 60 *Lactobacillus* sp. strains, including *L. pentosus* ŁOCK 0979, cultured in the presence of polyols and galactosyl-polyols as alternative carbon sources [13] to select bacteria with strong antagonistic properties against as many indicator fungal strains as possible. *L. pentosus* ŁOCK 0979 was selected for further research as one of the most prospective antifungal strain. In this context, the use of polyols and their galactosyl derivatives to enhance the inhibitory properties of lactobacilli is a novel solution which offers a promising method for modulating LAB metabolism. In situ studies on food products describing enhanced antifungal activity of lactobacilli on fruits have been presented by Lipinska et al. [13].

In the presented experiments, *L. pentosus* ŁOCK 0979 exhibited the ability to partially absorb lactitol, mannitol, and all the tested galactosyl-polyols. While Tyler et al. [30] isolated *Lactobacillus florum* 2F, a heterofermentative strain which can biosynthesize erythritol and mannitol, the consumption of polyols and galactosyl-polyols by *Lactobacillus* bacteria represents a new line of research with scant available literature.

Since the antifungal activity of lactobacilli consists of a complex set of interactions beginning at the cellular level, in this study considerable attention was given to the enzymatic activity of the bacteria in the presence of polyols and gal-polyols. Some small differences were found in enzymes metabolizing lipids, proteins, and phosphates, and in particular in esterase and esterase lipase, which catalyze the hydrolysis and synthesis of organic acid esters, primarily from water-soluble substrates, such as triacylglycerols containing short chain fatty acids. Bacterial esterase activity promotes the hydrolysis of a wide spectrum of substrates to acids [5, 28], including antifungal metabolites. LAB enzymes can be used to modify the gustatory and olfactory properties of wines and cheeses and to produce some ingredients of foodstuffs, pharmaceuticals, and cosmetics [12]. Fatty acids and hydroxy fatty acids synthesized by LAB affect fungal viability by irreversibly weakening and deforming the lipid bilayer [23]. The morphological changes in the structure of the cell walls of *C. vini* (strains 0008 and 0009) and *A. brassicicola* mycelia, which were revealed in this study using SEM and light microscopy, corroborate that mechanism of action for the obtained CFSs, which led to strong deformation of yeasts’ surface; similar morphological changes of *Candida* sp. were described by Shengli et al. [27]. In the presence of CFSs, yeasts can produce extracellular matrix to promote their adherence and protect cells from environmental insults [29].

## Conclusions

The antifungal activity of *L. pentosus* ŁOCK 0979 depends on the bacterial culture medium as well as on the fungal strain. The present study shows changes in the antifungal profile of the studied bacterial strain linked to the composition of the culture medium. Although no single decisive factor (metabolite) was found to be responsible for inhibiting fungal growth, the results indicate how bacterial metabolite profiles may be beneficially modulated. Thus, the authors have broken new ground in developing natural ways of ensuring the microbiological safety of food.
